# Invasive Meningococcal Capsular Group Y Disease, England and Wales, 2007–2009

**DOI:** 10.3201/eid1801.110901

**Published:** 2012-01

**Authors:** Shamez N. Ladhani, Jay Lucidarme, Lynne S. Newbold, Stephen J. Gray, Anthony D. Carr, Jamie Findlow, Mary E. Ramsay, Edward B. Kaczmarski, Raymond Borrow

**Affiliations:** Health Protection Agency, London, UK (S.N. Ladhani, M.E. Ramsay);; Health Protection Agency, Manchester, UK (J. Lucidarme, L.S. Newbold, S.J. Gray, A.D. Carr, J. Findlow, E.B. Kaczmarski, R. Borrow);; University of Manchester, Manchester (R. Borrow)

**Keywords:** Meningococcal, capsular group Y, vaccine, lpxL1, NadA, outcome, bacteria, England, Wales

## Abstract

Enhanced national surveillance for invasive meningococcal disease in England and Wales identified an increase in laboratory-confirmed capsular group Y (MenY) disease from 34 cases in 2007 to 44 in 2008 and 65 in 2009. For cases diagnosed in 2009, patient median age at disease onset was 60 years; 39% of patients had underlying medical conditions, and 19% died. MenY isolates causing invasive disease during 2007–2009 belonged mainly to 1 of 4 clonal complexes (cc), cc23 (56% of isolates), cc174 (21%), cc167 (11%), and cc22 (8%). The 2009 increase resulted primarily from sequence type 1655 (cc23) (22 cases in 2009, compared with 4 cases each in 2007 and 2008). cc23 was associated with *lpxL1* mutations and meningitis in younger age groups (<25 years); cc174 was associated with nonmeningitis, particularly pneumonia, in older age groups (>65 years). The increase in MenY disease requires careful epidemiologic and molecular monitoring.

## Abstract 2

Increases may result from mutations that allow the organism to evade the immune system.

Invasive meningococcal disease is associated with substantial rates of illness and death worldwide (*1*), with most infections caused by 5 capsular groups, namely A, B, C, W135, and Y (*2*). In the United Kingdom, where capsular group C (MenC) conjugate vaccines have been routinely used since 1999 (*3**,**4*), capsular group B (MenB) causes >80% of confirmed cases, particularly among infants and adolescents (*5*). Invasive infections caused by other capsular groups are infrequent and sporadic (*6**,**7*).

Invasive capsular group Y (MenY) has historically been uncommon in the United Kingdom and until recently accounted for <30 cases annually (*5*). After 2006, however, enhanced surveillance by the Health Protection Agency (HPA) identified an increase in invasive MenY cases in England and Wales. In addition, recent surveys suggest that MenY carriage in England increased substantially during the past decade (*8**,**9*). Consequently, the HPA investigated the clinical, epidemiologic, and microbiologic characteristics of invasive MenY disease in England and Wales during 2007–2009.

## Methods

The HPA conducts meningococcal surveillance in England and Wales through a combination of clinical and laboratory reporting schemes and receives all meningococcal death registrations from the Office for National Statistics (www.statistics.gov.uk). In addition, the HPA Meningococcal Reference Unit provides a national service for species confirmation and grouping/typing of invasive *Neisseria meningitidis* and offers free nonculture confirmation by using PCR directly from clinical specimens routinely submitted by National Health Service hospital laboratories (*5*). Data from all sources are reconciled in a single database and deduplicated regularly. Invasive meningococcal disease was defined as isolation of the organism or identification of meningococcal DNA from a normally sterile body site. MenY was confirmed by sero/genogrouping (*5*).

### Clinical Isolates

Isolates were preserved at –80°C on Microbank cryovials (Pro-Lab Diagnostics, Richmond Hill, Ontario, Canada). Overnight cultures were performed on Columbia agar plus 5% (vol/vol) horse blood (Oxoid, Basingstoke, UK) at 37°C in an atmosphere containing 5% CO_2_.

### Bacterial Genomic DNA Extraction

DNA was extracted by using the DNeasy blood and tissue kit (QIAGEN, Crawley, UK). Briefly, 1 mL meningococcal suspension in physiologic saline (adjusted to an optical density equal to 0.1 at 650 nm) was heat-killed at 60°C for 70 min and then pelleted at 6,000 × *g* for 10 min. After aspiration of the supernatant, DNA was extracted by using the manufacturer’s gram-negative protocol (QIAGEN) (*10*).

### Molecular Typing

Multilocus sequence typing and genotypic analysis of PorA variable regions (VRs) (*porA* VR1 and VR2), ferric enterochelin receptor VR (*fetA*), neisserial heparin-binding antigen (*nhba*), neisserial adhesin A (*nadA*), and factor H binding protein (*fhbp*) encoding regions were performed by using described protocols (*11**,**12*). Molecular typing results are reported by using the following recommended format: capsular group: porA (P1) subtype.VR1,VR2: fetA VR: ST- (cc); for example, Y: P1.5-1,10-4: F4-1: ST-23 (cc23) (*13*). Sequence data have been deposited in GenBank under the following accession numbers (corresponding peptide subvariant identifiers in parentheses): for *nhba*, JN166971 and JN166972 (P0006), JN166973 (P0007), JN166974 (P0008), JN166975 (P0009), JN166976 (P0020), JN166977 (P0024), and JN166978 (P0242); and for *nadA*, JN166979 (P0008). *fhbp* subvariants are assigned in the format “Novartis variant family.peptide” according to www.pubmlst.org.

The lauroyl acyltransferase gene, *lpxL1*, was genetically characterized by using primers listed in Table 1 (*14*). *lpxL1* PCRs were performed by using the HotStarTaq DNA polymerase kit (QIAGEN), with an initial activation step of 96°C for 15 min and a final step of 72°C for 7 min. The default PCR used primers *lpxL1-F* and *lpxL1-R* in which a single reaction comprised 2.5 µL of 10× PCR buffer (provides 1.5 mmol/L MgCl), 2.5 µL of each primer (5 µmol/L stock), 0.5 µL dNTP mixture (10 mmol/L [per dNTP] stock), 0.125 µL HotStar Taq (0.625 units), 14.875 µL molecular-grade water, and 2 µL of DNA template. Thermocycling conditions comprised 35 cycles of 96°C for 30 s, 63°C for 30 s, and extension at 72°C for 60 s. Where the default PCR was unsuccessful, further PCR was performed by using the alternative reverse primer, *lpxL1-rR4*, in conjunction with a final MgCl_2_ concentration of 1.875 mmol/L and an extension step of 200 s. Sequence analysis was performed by using the BigDye Terminator v3.1 Cycle Sequencing Kit (Applied Biosystems, Foster City, CA, USA). A single sequencing reaction comprised 1.75 µL 5× sequencing buffer, 0.5 µL BigDye master mix, 0.66 µL primer (5 mmol/L stock), 6.09 µL molecular-grade water, and 1 µL purified PCR product.

**Table 1:** Primers used for genotypic analysis of *lpxL1* of meningococcal capsular group Y, England and Wales, 2007–2009*

PCR/sequence	Primer identification no.	Direction	Sequence, 5′ → 3′	Reference
PCR/sequence	lpxL1-F†‡§	Forward	TGCAGGTCAAACAGGCGGTAGT	(*14*)
PCR/sequence	lpxL1-R†¶#	Reverse	TTCAT(A/G)GGTTTGCGGTATTTCTTCCA	(*14*)
PCR	lpxL1-rR4‡	Reverse	TCCACTTGAAATCGCGGCTGTC	NA
Sequence	lpxL1-s1C#	Forward	GTTCGAGATGGCGGTGTAC	NA
Sequence	lpxL1-s2#	Reverse	GAATCGTTGCGTCCGAAATCCTG	NA
Sequence	lpxL1-rR3§	Reverse	AATACAGGCTTTCGCCTGCG	NA
Sequence	lpxL1-Rnew§	Reverse	GTCAGTAAAAATCGGGGCTGCC	NA

*NA, not applicable. †Default PCR primer. ‡Alternative PCR primer. §Used for sequence analysis of alternative PCR products (*14*). ¶Degenerate base added for this study. #Used for sequence analysis of default PCR products.

### Clinical Follow-up

Clinical follow-up was conducted for all MenY cases in 2009 confirmed by culture, PCR, or both. The general practitioner, hospital physician, or local health protection unit was contacted for information about underlying medical conditions, recent travel, clinical presentation, sequelae, and outcome.

### Statistical Methods

Data were analyzed by using Stata version 11.0 (StataCorp LP, College Station, TX, USA). Annual age-specific population estimates were obtained from the Office for National Statistics. Data that did not follow a normal distribution were described as medians with interquartile ranges (IQRs) and compared by using the Mann-Whitney U test. Proportions were compared by using the χ^2^ test or Fisher exact test, as appropriate. Logistic regression was used to calculate odds ratios (ORs) with 95% CIs after adjustment for potentially confounding variables and to identify independent risk factors for death.

### Ethical Approval

HPA has approval under the Health and Social Care Act 2001 to process confidential patient information for public health purposes. Details are available at www.legislation.hmso.gov.uk/si/si2002/20021438.htm.

## Results

### Epidemiology

**Figure 1:**
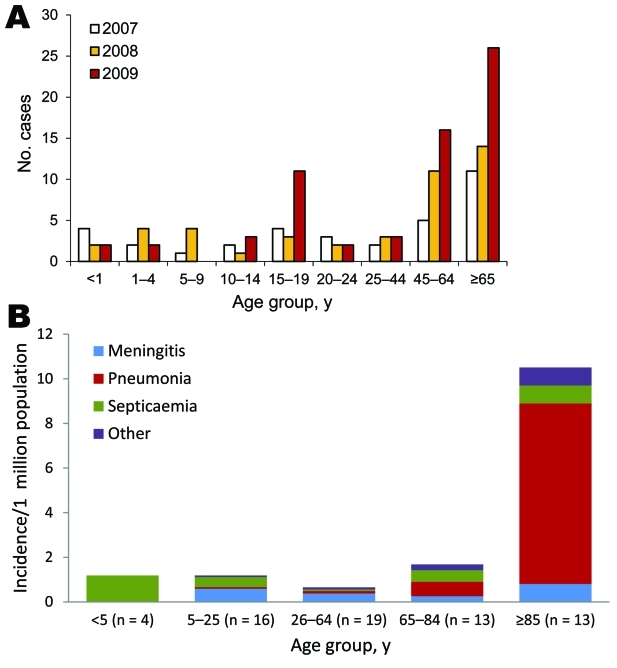
Number of persons with invasive meningococcal capsular group Y (MenY), by age group and year (A) and incidence with clinical features of MenY disease, by age group, in 2009 (B), England and Wales.

Invasive meningococcal disease in England and Wales declined from 1,283 cases in 2007 to 1,228 in 2008 and 1,031 in 2009, with 54%, 53%, and 57% of cases, respectively, confirmed by PCR alone. MenB cases accounted for 1,096 (85%), 1,102 (90%), and 917 (89%) cases during this period, and MenY cases increased from 34 (3%) to 44 (4%) and 65 (6%), higher than at any time during the previous 30 years (*5**,**15*) (Figure 1, panel A). Surveillance and diagnostic methods that might have contributed to this increase did not change.

### Clinical Follow-up

In 2009, there were 65 invasive MenY cases (46 culture-confirmed and 19 PCR-confirmed), equivalent to 1.2 cases per million population (Table 2). The incidence was >10-fold higher for persons >85 years of age (10.5/1,000,000) than for persons <85 years of age (0.99/1,000,000). Median age at disease onset was 59.9 years (IQR 19.2–82.5 years). Most cases (58 [89%]) occurred among whites; the remainder occurred among Indians (4 [6%]) and black Afro-Caribbeans (3 [5%]). Clinical features varied by age group (Table 2; Figure 1, panel B). More than one third (25 [38%]) had at least 1 underlying medical condition; 12 (18%; 95% CI 9.9%–30.0%) died. Likelihood of underlying medical conditions and death increased with age. Case-fatality rate (CFR) was significantly higher for persons with underlying medical conditions (11 [44%] of 25 vs. 1 [3%] of 40; χ^2^ = 17.6, p<0.001) and varied by clinical feature, being substantially higher for pneumonia (9 [47%] of 19) than for meningitis (2 [9%] of 22), septicemia (1 [6%] of 17), or other diagnoses (no deaths). Median age at disease onset was higher for persons with pneumonia than for those with other conditions (86.1 years [IQR 69.9–90.0] vs. 42.8 years [IQR 14.4–71.1]; p<0.0001), and persons with pneumonia were also more likely to have underlying medical conditions (13 [68%] of 19 vs. 12 [26%] of 46; χ^2^ =10.2, p = 0.001). After adjustment for age, underlying medical condition (OR 19.2, 95% CI 2.1–174, p = 0.009) and pneumonia (OR 7.0, 95% CI 1.4–36.4, p = 0.020) remained independently associated with death.

**Table 2:** Clinical presentation of, risk factors for, and outcome of patients with invasive meningococcal capsular group Y disease, England and Wales, 2007–2009

Variable	Age group, y, no. (%) patients				Total, n = 65
	<25, n = 20	25–64, n = 19	65–84, n = 13	>85, n = 13	
Female sex	11 (55)	14 (74)	10 (77)	12 (92)	47 (72)
Travel*	2 (10)	2 (11)	1 (8)	0	5 (8)
Clinical feature					
Meningitis	8 (40)	11 (58)	2 (15)	1 (8)	23 (35)
Pneumonia	1 (5)	3 (16)	5 (39)	10 (77)	19 (29)
Septicemia	10 (50)	2 (11)	4 (31)	1 (8)	17 (26)
Other	1 (5)	3 (16)	2 (15)	1 (8)	7 (11)
Underlying conditions†	3 (15)	5 (26)	7 (54)	10 (77)	25 (39)
Immune deficiency‡	2 (10)	1 (5)	2 (15)	3 (23)	8 (12)
Sequelae§	1 (5)	1 (5)	0	1 (8)	3 (5)
Deaths	2 (10)	1 (5)	2 (15)	7 (54)	12 (19)

*Travel-associated infection included 2 visitors from Australia and South America and 3 residents who had traveled to Jamaica and Thailand and taken a Mediterranean cruise in the preceding 4 weeks. †Underlying medical conditions included complement deficiency (1 person), diabetes mellitus (1), and systemic lupus erythematosus among persons <25 years of age; chronic liver disease (3), diabetes mellitus (1), and rheumatoid arthritis on immunosuppressive therapy (1) among persons 25–64 years of age; malignancy undergoing chemotherapy (2), diabetes mellitus (2), chronic heart disease (2), and chronic respiratory disease (1) among persons 65–84 years of age; and chronic heart disease (7), malignancy undergoing chemotherapy (2), chronic respiratory disease (1), and rheumatoid arthritis on immunosuppressive therapy (1) among persons >85 years of age. ‡Immunodeficiency included malignancy receiving chemotherapy (4 persons), autoimmune disease requiring immunosuppressant therapy (3), and complement deficiency (1). §Sequelae included hemiparesis after meningitis in a toddler, bilateral sensorineural deafness requiring cochlear implantat after meningitis in an adult, and chronic renal insufficiency in an elderly person with septicemia.

### Molecular Typing

All 114 invasive MenY isolates for 2007–2009 were subjected to molecular typing. Age distribution was as follows: 21 (18%) cases among persons <5 years, 42 (37%) among persons 5–64 years, and 51 (45%) among persons >65 years of age. Clinical diagnosis was available for 60 cases (46 culture-positive cases from 2009 and 14 cases from the previous 2 years for which clinical details were provided with the isolate) and included meningitis, septicaemia, and pneumonia (18 [30%] each) and other presentations (6 [10%]). Almost all (110 [96%]) isolates belonged to 1 of 4 ccs: cc23 (64 [56%]), cc174 (24 [21%]), cc167 (12 [11%]), and cc22 (9 [8%]) (Table A1). The only notable increase was observed for cc23 isolates, which more than doubled from 14 and 16 in 2007 and 2008, respectively, to 34 in 2009.

One cc23 isolate was not fully characterized by sequence type (ST) because existing primers failed to amplify the glucose-6-phosphate dehydrogenase gene (*gdh*) locus. The remaining 6 loci were successfully characterized and sufficient to assign the isolate to cc23. Of the 63 remaining cc23 isolates, 52 (83%) constituted ST-1655 (30 [48%]) or ST-23 (22 [35%]), and 20 (83%) of 24 cc174 isolates belonged to ST-1466 (Table A1). The 2009 increase resulted almost exclusively from expansion of ST-1655, from 4 (13%) and 4 (12%) cases in 2007 and 2008, respectively, to 22 (44%) in 2009. Other STs accounted for 27, 29, and 28 cases during these 3 years. We found no evidence of temporospatial clustering of ST-1655 cases. All ST-1655 isolates had *porA* VR1 P1.5-1 and *fetA* F4-1 and varied only in their *porA* VR2 region; clone Y:P1.5-1,10-1:F4-1:ST1655 (cc23) was the most prevalent (21 [70%] of 30) and responsible for 3 cases each in 2007 and 2008 and 15 cases in 2009. Cases from Y:P1.5-1,10-4:F4-1:ST1655 (cc23) increased to a lesser extent from 0 and 1 to 5 cases during the 3 years, respectively.

#### PorA

Genotypic analysis of *porA* VR1 showed that almost two thirds of the 114 strains belonged to P1.5-1 (72 [63%]), followed by P1.21 (16 [14%]) and P1.5-2 (12 [11%]). *porA* VR2 variants included mainly P1.10-1 (31 [27%]), P1.10-4 (20 [18%]), P1.2-2 (19 [17%]), and P1.16 (16 [14]%) (Table A1).

#### FetA

We found 3 *fetA* types—F4-1 (48 [42%]), F3-7 (23 [20%]), and F5-8 (16 [14%])—in >75% of strains (Table A1). F4-1 belonged mainly to cc23 (45 [94%] of 48 cases), and F3-7 variants all belonged to cc174.

#### NadA

The *nadA* gene was found exclusively in cc174 isolates, all 24 of which harbored the gene. Sequence analysis of *nadA* was performed among a subset of cc174 isolates (collected during epidemiologic year 2007–08 [7 isolates]) all harbored identical alleles encoding NadA peptide subvariant 3.P0008.

#### lpxL1

**Figure 2:**
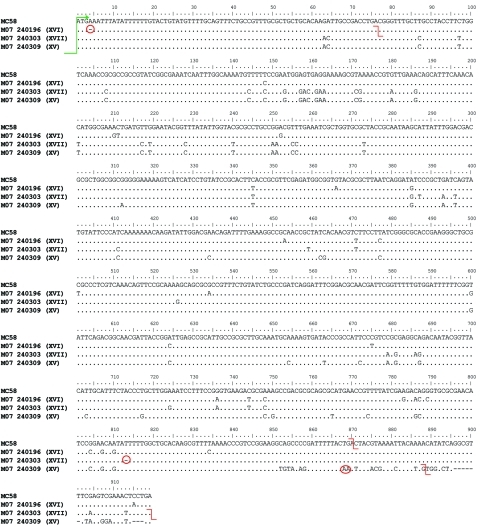
Newly identified *lpxL1* mutations XV, XVI, and XVII. *lpxL1* sequence data from isolates harboring each of the corresponding meningococcal capsular group Y mutations (in parentheses), England and Wales. Mutations are aligned with the full-length gene from strain MC58. All of the alleles share a common start codon (green arrow). Mutations and stop codons are denoted by red circles and red lines, respectively. Mutation XVI is a single-base deletion at nt A_4_ that causes a frame shift resulting in a premature stop codon at nt 74. Mutation XVII is a single base deletion at nt T_813_. This causes a frame shift resulting in a late stop codon at nt 919. Mutation XV is a polymorphism at the region of the prototypical stop codon (that of strain MC58). In affected isolates, 3 adenines occupy the site that comprises the stop codon, TGA, in MC58. The next available stop codon occurs 15 bp downstream. The encoded peptide has an additional 6 aa.

*lpxL1* genotype was indeterminate in 1 cc174 strain because the existing primers failed to amplify the gene and flanking regions, possibly because of variation in the primer target sites. Mutations in the *lpxL1* gene were identified in all but 2 cc23 isolates and none of the other ccs. Of the 62 cc23 isolates with an *lpxL1* mutation, all 30 ST-1655 isolates harbored a previously unpublished single-base deletion at nt A_4_ (designated mutation XVI) (Figure 2), which resulted in a frame shift and premature stop codon. This mutation also was observed in 1 ST-8414 (cc23) and 3 ST-23 (cc23) isolates. Fifteen of the remaining 19 ST-23 isolates harbored frame-shifted alleles (1 mutation IV, 13 mutation V, and 1 mutation VI [*15*]), whereas another was interrupted by the insertion sequence *IS1301* (mutation I) (*16*). The remaining 3 ST-23 isolates harbored an *lpxL1* allele in which 3 adenines occupied the prototype (strain MC58) stop codon. This configuration was designated mutation XV. The resultant allele was extended by 18 bp. Of the remaining 9 cc23 isolates (1 not fully characterized for ST and the remainder belonging to other less well-represented STs), another 2 harbored mutation XV, 6 harbored mutation V (*16*), and 1 harbored a previously unpublished deletion at nt T_813_ (designated mutation XVII). The frame-shifted allele was extended by 47 bp.

Approximately half (n = 56) of the isolates had mutations, which potentially resulted in an underacylated lipid A moiety of lipooligosaccharide and belonged mainly to either ST-1655 (30 [54%]) or ST-23 (18 [32%]). The number of mutant *lpxL1* isolates increased from 12 each in 2007 and 2008 to 32 in 2009, mainly because of the increase in ST-1655.

Mutation XV is predicted to extend the encoded peptide product by 6 aa. However, it does not alter the upstream amino acid sequence, which remains complete in comparison with the putative wild-type peptide. It is possible, therefore, that the enzyme encoded remains functional. Of the affected isolates, 3 of 5 occurred in children <15 years of age, none of whom died; clinical diagnosis was available for 4 case-patients, including 3 with septicemia and 1 with meningitis; case numbers did not increase with time (1 in 2007, 2 in 2008, and 2 in 2009).

### Factor H–Binding Protein

Almost all isolates (108 [95%]) harbored *fhbp* variant 2 (subfamily A) alleles. Three (3%) isolates harbored *fhbp* variant 1 (subfamily B) alleles encoding fhbp peptide variants 1.13 (cc174), 1.258 (cc174), and 1.335 (cc23); all 3 isolates caused disease in adults with no fatalities). Three (3%) isolates harbored *fhbp* variant 3 (subfamily A) alleles (encoding fhbp peptide variants 3.293 [2 isolates] and 3.300 [1 isolate]; all isolates belonged to cc23 and caused disease in children with no fatalities) (Table A1).

### Neisserial Heparin–Binding Antigen

All MenY isolates harbored *nhba* alleles (Table A1). In terms of the NHBA peptide subvariants encoded, cc22 and cc167 were exclusively represented by P0020 and P0009, respectively. cc23 was predominantly represented by P0007 (43 [67%] of 64) as well as P0008 (14 [22%]) and P0006 (7 [11%]); cc174 was represented predominantly by P0006 (23 [96%] of 24 isolates).

### MenY Clones

The 5 most prevalent MenY clones were Y:P1.5-1,10-1: F4-1:ST1655 (cc23) (21 [18%]); Y:P1.21,16:F3-7: ST-1466 (cc174) (13 [11%]); Y:P1.5-1,2-2:F5-8:ST23 (cc23) (11 10%]); Y:P1.5-1,10-4:F4-1: ST-1655 (cc23) (6 [5%]); and Y:P1.5-1,10-4:F4-1: ST-23 (cc23) (4 [4%]). All 5 deaths in persons infected with the 3 most prevalent clones occurred among those with pneumonia. No deaths occurred among persons infected with the remaining 2 clones.

### Association with Age, Clinical Features, and Outcome

cc23, which harbored all the *lpxL1* mutations, was associated with younger age (<25 years) at disease onset (30 [47%] of 64 cc23 isolates vs. 9 [18%] of 50 other isolates; χ^2^ = 10.4, p = 0.001) and with meningitis (16 [40%] of 40 vs. 2 [10%] of 20; χ^2^ = 5.7, p =0.017) but not with death (9 [14%] of 64 vs. 6 [12%] of 50; χ^2^ = 0.10, p = 0.75). In contrast, cc174, all of which had the *nadA* gene, was associated with older age (>65 years) at onset (17 [71%] of 24 cc174 isolates vs. 34 [38%] of 90 other isolates; χ^2^ = 8.4, p = 0.004) and lack of meningitis (9 [100%] of 9 vs. 33 [65%] of 51; χ^2^ = 4.5, p = 0.033), with a trend toward presence of pneumonia (5 [56%] of 9 vs. 13 [25%] of 51; χ^2^ = 3.3, p = 0.070) but not death (3 [13%] of 24 vs. 12 [13%] of 90; χ^2^ = 0.011, p = 0.92). Of the 114 genotypically characterized isolates, CFR was 13% (15 deaths) and was not associated with any particular cc: 14% (9 deaths) for cc23; 13% (3 deaths) for cc174; and 8%, 11%, and 20% (1 death each) for cc167, cc22, and the remaining ccs, respectively.

## Discussion

Routine provision of a national reference service by HPA for meningococcal species confirmation, capsular grouping and typing, and regular integration of multiple data sources provides a robust and stable enhanced national surveillance for invasive meningococcal disease covering the 55 million persons in England and Wales. Our surveillance indicates an increase in invasive MenY cases in England and Wales since 2007. A similar increase was observed a decade ago in the United States, where MenY currently accounts for one third of all meningococcal infections (*17*). Clinical follow-up of cases diagnosed in 2009 indicated that MenY affects mainly older adults with underlying medical conditions (*18**,**19*). Almost one third of MenY case-patients had pneumonia. MenY is the most prevalent cause of meningococcal pneumonia (*18*), although capsular group W135 pneumonia also has been described (*20**–**22*), whereas MenB and MenC rarely cause pneumonia (*18*). In 1 study, pneumonia was 4× more common among MenY cases (12%) than among cases with other capsular groups (3%), even after adjustment for patient age (*23*). Similarly, a literature review of 58 published cases of meningococcal pneumonia during 1974–1998 found MenY as the most common pathogen (44%) and caused disease mainly among older adults with underlying medical conditions (*18*). CFR for MenY pneumonia was 8.6% (*18*), which was similar to other MenY reports (*5**,**23**–**28*). The higher CFR in our surveillance (18% overall and 47% for pneumonia) may be explained by the older age of pneumonia case-patients and higher prevalence of underlying medical conditions. CFR was also higher than that reported for MenB (5.1%) or MenC (11.6%) in England and Wales (*5*).

Molecular typing showed that ST-1655 (cc23) was responsible for the increase in MenY cases. We found no evidence of clustering of cases to suggest an outbreak. A recent meningococcal carriage study involving first-year university students in Nottingham, United Kingdom, during 2008–09 reported rates of 42% at the initial time-point, which increased to 62% during the study, with more than half the carriage isolates belonging to MenY (*8*). These rates are substantially higher than those in a previous multicentre study among 15–19-year-old UK school students during 1999–2001, where MenY carriage was only 5%–6% (*9*). In the Nottingham study, more than half of the carriage isolates belonged to 1 of 4 *porA* types (P1.5-1,10; P1.21,16; P1.5,2; and P1.21-7,16) and 4 ccs (cc23, cc60, cc167, and cc174) (*8*), which confirms that most meningococcal strains causing invasive disease—particularly ST-1655 (cc23)—also are circulating in England.

The association between MenY ccs and specific clinical features in different age groups is intriguing. Certain strains might have acquired specific virulence factors that facilitate invasion of particular organs, such as the lungs or the brain. In our study, for example, infection with MenY strains harboring *lpxL1* mutations was associated with meningitis, particularly in younger persons. In contrast, cc174 strains, which all harbored *NadA*, were more likely to cause pneumonia in older adults.

Meningococcal lipid A is the bioactive component of lipooligosaccharide (or endotoxin) that induces a proinflammatory cytokine response through the toll-like receptor 4 (TLR4) innate immunity pathway (*29*). Up to 9% of invasive clinical meningococcal strains (including MenY) have 5 instead of 6 acyl chains because of inactivating mutations in the acyl-transferase gene, *lpxL1* (*16*). Such mutant strains induce a lower proinflammatory cytokine response, which may enable them to evade the host innate immune system (*16**,**30*). They were also more likely to cause meningitis in younger persons and less likely to have features associated with activation of the coagulation system in meningococcal septicemia (rash, septic shock, or thrombocytopenia) (*16*). A recent study in the Netherlands reported a high proportion (61%) of *lpxL1* mutations among 71 cc23 carriage isolates (predominantly MenY) compared with 0.3% among 751 non-cc23 carriage isolates and 8.6% among 464 non-cc23 invasive disease isolates (*31*). No cc23 strains existed among invasive clinical isolates collected in the Netherlands during 2001–2006 (*31*).

In our study, *lpxL1* mutations were identified in 97% of invasive cc23 MenY isolates and occurred infrequently among non-cc23 MenY isolates. The 2009 increase in ST-1655 (cc23), which all harbored *lpxL1* mutations, is concerning because of its preponderance for causing meningitis in healthy young persons. Many of the *lpxL1* mutations result from potentially reversible alterations in homopolymeric tracts, and phase variation of *lpxL1* has been speculated to occur within the host to avoid enhanced recognition by the immune system after crossing the nasopharygeal epithelium (*16*). The uniquely high frequency of mutants among invasive disease and carriage cc23 isolates suggest that these have an enhanced propensity to the mutant state for reasons as yet unknown. The diversity of the mutations within closely related lineages suggest that they have occurred in independent events. The most widely distributed mutation among cc23 (mutation V) occurs in a relative long poly-A tract, which would be more likely to occur independently at relatively high frequencies. Mutation XVI occurs in a relatively short poly-A tract, as reflected in its relatively narrow distribution. That it is the sole mutation among ST-1655 isolates probably reflects the recent, rapid expansion of this particular lineage. We also have identified new *lpxL1* mutations that could potentially result in underacylated lipid A, but whether the mutations result in underacetylation will require confirmation in future studies.

The *nadA* gene was present only in cc174 strains, representing 21% of MenY isolates. A similar proportion (23%) was reported among invasive MenB isolates in England and Wales (*12*), but may be present in up to 100% of certain hypervirulent lineages (*32**,**33*). NadA is a surface-exposed neisserial adhesin that binds human β1-intergrins (*34*) and is involved in epithelial cell adhesion and invasion (*32**,**33*). Interactions between bacterial adhesins and β1 integrins have been described for several pathogens that invade the respiratory or gastrointestinal mucosa and play a major role in mucosal translocation and triggering the release of chemokines (*34*). NadA may thus enable MenY strains to invade locally and cause respiratory tract infections, particularly among elderly persons, who often have multiple underlying medical conditions. We have also reported on other meningococcal antigens that are relatively conserved within certain ccs. Some of these antigens are included in investigational meningococcal vaccines currently undergoing clinical trials. Licensed meningococcal quadrivalent conjugate vaccines are available, but given the small number and age distribution of patients, routine vaccination against these capsular groups is unlikely to be cost effective at this time.

Invasive MenY disease has increased in England and Wales, mainly because of ST-1655 (cc23). This cc was significantly more likely to cause meningitis, mainly in young adults, and was associated with the presence of *lpxL1* mutations. The increase in invasive MenY disease, particularly as accompanied by recent reports of substantially increased carriage of MenY, will require careful epidemiologic and molecular monitoring.

## Appendix

**Table A1:** Molecular analysis of 114 MenY isolates causing invasive disease, England and Wales, 2007–2009*

Clonal complex	Sequence type	*fhbp*	*nhba*	*FetA*	*PorA*	*lpxL1* mutation
23 (64)†	1655 (30)	2.25 (30)	P0007 (30)	4-1 (30)	P1.5-1,10-1,36-2 (18)	XVI (18)
					P1.5-1,10-4,36-2 (6)	XVI (6)
					P1.5-1,10-10,36-2 (3)	XVI (3)
					P1.5-1,10-1,25 (2)	XVI (2)
					P1.5-1,10-12,36-2 (1)	XVI (1)
	23 (22)	2.25 (20)	P0006 (5)	5-8 (5)	P1.5-1,2-2,36-2 (5)	XV (3)
						IV (1)
						V (1)
			P0007 (7)	4-1 (7)	P1.5-1,10-4,36-2 (4)	XVI (3)
						V (1)
					P1.5-2,10-1,25 (1)	V (1)
					P1.5-2,10-1,36-2 (2)	V (1)
						VI (1)
			P0008 (8)	5-8 (7)	P1.5-1,2-2,36-2 (5)	V (5)
					P1.5-1,2-2,25 (1)	V (1)
					NP (1)	V (1)
				1-7 (1)	P1.5-1,2-2,36-2 (1)	V (1)
		2.104 (1)	P0008 (1)	2-13 (1)	P1.5-2,10-2,104 (1)	I (1)
		3.300 (1)	P0007 (1)	4-1 (1)	P1.5-2,10-1,36-2 (1)	V (1)
	183 (2)	2.104 (2)	P0008 (2)	NP (1)	P1.5-2,10-2,36-2 (1)	Intact (1)
				4-1 (1)	P1.5-2,10-2,36-2 (1)	V (1)
	8411 (2)	2.25 (2)	P0006 (2)	5-8 (2)	P1.5-1,2-2,36-2 (2)	XV (2)
	Other (7)	2.25 (4)	P0007 (3)	4-1 (3)	P1.5-1,10-4,36-2 (1)	V (1)
					P1.5-1,10-4,36-2 (1)	XVI (1)
					P1.5-2,10-1,36-2 (1)	V (1)
			P0008 (1)	5-8 (1)	P1.5-1,2-2,36-2 (1)	V (1)
		2.104 (1)	P0008 (1)	2-13 (1)	P1.5-2,10-2,36-2 (1)	XVII (1)
		3.293 (1)	P0007 (1)	4-1 (1)	P1.5-2,10-1,36-2 (1)	V (1)
		1.335 (1)	P0008 (1)	4-1 (1)	P1.18-1,25,38-1 (1)	Intact (1)
	NK (1)	3.293 (1)	P0007 (1)	4-1 (1)	P1.5-2,10-1,36-2 (1)	V (1)
174 (24)	1466 (20)	2.21 (18)	P0006 (17)	3-7 (16)	P1.21,16,37-1 (10)	Intact (10)
					P1.21,16,21 (1)	Intact (1)
					P1.22,9,35-1 (2)	Intact (2)
					P1.5-1,10-8,36-2 (2)	Intact (1)
						NK (1)
					P1.7,30,38 (1)	Intact (1)
				2-16 (1)	P1.5-1,2-2,36-2 (1)	Intact (1)
			P0242 (1)	3-7 (1)	P1.22,9,35-1 (1)	Intact (1)
		1.13 (1)	P0006 (1)	3-7 (1)	P1.21,16,37-1 (1)	Intact (1)
		1.258 (1)	P0006 (1)	3-7 (1)	P1.21,16,37-1 (1)	Intact (1)
	7850 (2)	2.21 (2)	P0006 (2)	3-7 (2)	P1.21,16,21 (1)	Intact (1)
					P1.21,16,37-1 (1)	Intact (1)
	8413 (1)	2.21 (1)	P0006 (1)	3-7 (1)	P1.22,9,35-1 (1)	Intact (1)
	7263 (1)	2.21 (1)	P0006 (1)	3-7 (1)	P1.21,16,37-1 (1)	Intact (1)
167 (12)	1627 (4)	2.23 (4)	P0009 (4)	3-6 (4)	P1.5-1,10-4,36-2 (2)	Intact (2)
					P1.5-1,10-8,36-2 (1)	Intact (1)
					P1.5-1,10-1,36-2 (1)	Intact (1)
	168 (3)	2.19 (2)	P0009 (2)	3-1 (1)	P1.5-1,10-4,36-2 (1)	Intact (1)
				5-2 (1)	P1.5-1,10-4,36-2 (1)	Intact (1)
		2.23 (1)	P0009 (1)	4-1 (1)	P1.5-1,10-8,36-2 (1)	Intact (1)
	1624 (3)	2.23 (3)	P0009 (3)	3-4 (3)	P1.5-1,10-4,36-2 (3)	Intact (3)
	7921 (1)	2.16 (1)	P0009 (1)	3-6 (1)	P1.5-1,10-4,36-2 (1)	Intact (1)
	8420 (1)	2.23 (1)	P0009 (1)	1-3 (1)	P1.5-1,10-1,36-2 (1)	Intact (1)
22 (9)	3651 (3)	2.25 (3)	P0020 (3)	5-1 (3)	P1.5-1,2-2,36-2 (2)	Intact (2)
					P1.5,2-2,36-2 (1)	Intact (1)
	114 (2)	2.16 (2)	P0020 (2)	5-1 (2)	P1.5-1,10-22,36-2 (2)	Intact (2)
	184 (1)	2.16 (1)	P0020 (1)	3-9 (1)	P1.18-1,3,38 (1)	Intact (1)
	1221 (1)	2.25 (1)	P0020 (1)	1-7 (1)	P1.18-1,3,38 (1)	Intact (1)
	2180 (1)	2.25 (1)	P0020 (1)	1-2 (1)	P1.18-1,3,38 (1)	Intact (1)
	2878 (1)	2.16 (1)	P0020 (1)	4-1 (1)	P1.18-1,3,38 (1)	Intact (1)
Unassigned (3)	3015 (2)	2.16 (2)	P0020 (2)	3-4 (1)	P1.18-1,3,38 (1)	Intact (1)
				5-8 (1)	P1.5-2,10-1,36-2 (1)	Intact (1)
	7786 (1)	2.16 (1)	P0020 (1)	3-4 (1)	NP (1)	Intact (1)
92 (1)	5418 (1)	2.21 (1)	P0009 (1)	4-1 (1)	P1.5-1,10-22,36-2 (1)	Intact (1)
103 (1)	103 (1)	2.25 (1)	P0024 (1)	3-9 (1)	P1.5-1,10-46,36-2 (1)	Intact (1)

*MenY, meningococcal capsular group Y; *fhbp* , factor H binding protein (Novartis variant family.peptide); *nhba*, neisserial heparin-binding antigen; *fetA*, ferric enterochelin receptor variable region; *porA*, PorA variable regions (P1.VR1,VR2,VR3); lpxL1, acyl-transferase gene; NK, not known; NP, no product. Numbers in parentheses are the number of isolates.

†The sequence type for 1 clonal complex 23 strain was not available because of failure of existing primers to amplify the glucose-6-phosphate dehydrogenase (*gdh*) locus, but the remaining 6 loci were successfully characterized and were sufficient to assign the isolate to clonal complex 23.
